# Electroencephalography (EEG) Reveals Increased Frontal Activity in Social Presence

**DOI:** 10.3390/brainsci11060731

**Published:** 2021-05-31

**Authors:** Anna Soiné, Alessandra Natascha Flöck, Peter Walla

**Affiliations:** 1 CanBeLab, Psychology Department, Webster Vienna Private University, Praterstrasse 23, 1020 Vienna, Austria; anna.soine@t-online.de (A.S.); aflock23@webster.edu (A.N.F.); 2 Faculty of Psychology, Sigmund Freud University, Sigmund Freud Platz 1, 1020 Vienna, Austria; 3 Faculty of Medicine, Sigmund Freud University, Sigmund Freud Platz 3, 1020 Vienna, Austria; 4 School of Psychology, Newcastle University, University Drive, Callaghan, NSW 2308, Australia

**Keywords:** EEG, social presence, anterior cingulate cortex, valence-specific activation, social neuroscience

## Abstract

It remains an unsolved conundrum how social presence affects the neural processes involved in adaptive situation-specific decision-making mechanisms. To investigate this question, brain potential changes via electroencephalography (EEG) and skin conductance responses (SCR) were taken within this study, while participants were exposed to pre-rated pleasant, neutral, and unpleasant pictures, which they had to rate in terms of their perceived arousal. Crucially, they had to—in respective runs—do this alone and in the presence of a significant other. Contrasting respective event-related potentials (ERPs) revealed significantly more negative going potentials peaking at 708 ms post stimulus onset at mid-frontal electrode locations (around FPz and AFz), when participants were exposed to neutral pictures while in the presence of a significant other. SCR results demonstrate higher states of arousal in the presence of a significant other regardless of picture emotion category. Self-reported arousal turned out to be highest in response to neutral pictures within the significant other condition, whereas in the alone condition in response to the pleasant pictures. In light of existing literature on social aspects and the anterior cingulate cortex (ACC), the ERP finding in the significant other condition, while rating emotionally neutral pictures, is interpreted as reflecting heightened ACC activation, which is supported by electrode locations showing significant brain activity differences as well as by source localization results. Neutral pictures are inherently ambiguous, and the current results indicate the presence of another person to change the way one processes, perceives, and acts on them. This is in support for theories proposing the ACC to be part of a larger signal-specification network that gauges relevant stimuli for adequate execution of control.

## 1. Introduction

Social presence alters the environmental circumstances that an individual is exposed to as well as their internal states. Consequently, it has an impact on human perception and hence situation-specific decision-making, especially since the costs of mistakes are often hypothesized to be higher when made in front of conspecifics than when alone [1,2,3]. To diminish the possibility of such costs, social cognition mechanisms incorporate a variety of mental processes, “ranging from perception to decision-making, underlying the ability to decode others’ intentions and behaviors to plan actions fitting with social and moral, besides individual and economic considerations” [4]. Being innate to human nature, the need for social connectedness aims towards long-term benefits for the concerned organism. In order to determine the most beneficial course of action in social presence, adaptive situation-specific decision-making is needed, as it is of vital significance to comprehend others’ affective and cognitive states as well as their resulting intentions [4]. This accurate estimate of the states of one’s social counterpart is important to construct a realistic automated preview of social circumstances, which is essential for adequately deciding between two or more alternative strategies in social presence [5,6].

The current study was designed to help solve the conundrum of how social presence affects the neural processes involved in adaptive situation-specific decision-making mechanisms. This shall be achieved by examining the affective component of differently valenced stimuli on human brain activation as measured via electroencephalography (EEG), subjective perception of arousal, and skin conductance response (SCR) during the presence of a significant other compared to being alone.

As mentioned in several studies, subcortical affective processing underlying the formation of feelings and emotions is mostly unavailable to articulation [7], while still representing an integral part of (social) situation-specific decision-making. It has been found that affective responses also trigger activation within a network of brain regions incorporating the anterior cingulate cortex (ACC) and the medial prefrontal cortex (mPFC) [8]. While EEG is not sensitive to subcortical brain processes it is sensitive to processes mediated by cortical structures including the ACC and the mPFC.

Given the manifold assumptions concerning the role of the ACC in affective processing and decision-making [1,5,8,9,10,11,12,13,14,15,16,17,18,19,20,21], a thorough illumination best achievable by a multiplicity of tools, which investigate cortical, sympathetic arousal, as well as subjective conscious appraisal and conscious thought (feelings), is required. To achieve this purpose, brain activity measured with EEG and SCR directly mirror affective processing referring to “neural activity representing the most basic decision-making quality that guides human behavior” [7]. In situation-specific decision-making mechanisms independent of the social context, affective processing accounts for the evaluation of how something is, an aspect which is hypothesized to be of un- or subconscious nature. Within this context, EEG measurements depict states of cortical arousal, while SCR data represent a measurement of sympathetic nervous system arousal. Self-report data, in turn, are treated as a direct function of conscious appraisal and conscious thought, stemming from the behavioral expression of feelings that originate within the very same affective processing signals (within this interpretation, emotions are understood as behavioral expressions of feelings, which in turn stem from affective processing signals. The intention and evolutionary purpose of emotions is to communicate affective feelings to others. Cognitive processing on the contrary is based on neural activity that codes for semantic aspects of information and is involved in the interpretation of what something is [7]).

Based on existing findings of neuroimaging studies about heightened ACC activation in the presence of aversive signals, we hypothesize to find increased activation (i.e., increased negative potentials) at electrode locations associated with ACC activity (mid-frontal locations). Within the context of affective processing in social situations, this activation pattern is expected to occur in response to negative pictures for the ACC’s role in negative affect and experienced as well as observed pain [9,22]. Further, a similar neural activity is likely to be detected in response to neutral pictures, as the ACC is indicated to be involved in several qualities of uncertainty and ambiguity processing [23,24]. In the search for the best value option between alternative strategies, such activation patterns are thought to be reflective of an individual’s ambiguity attitudes [5,24]. Grounded in established findings, a substantive relationship is assumed to exist between cerebral cortical activity as measured with EEG and sympathetic arousal as measured through skin conductance levels (SCL) [25]. In comparison to tonic SCL, SCR reflects more acute physiological indices responsible for adaptive bodily action in reaction to the presentation of emotion-related stimuli [26]. Given its usefulness as an informative indicator of autonomic arousal, we anticipate increases in frontal brain activity to be connected to sympathetic states measured with SCR [25].

## 2. Materials and Methods

### 2.1. Participants

We sampled 30 healthy, English speaking, right-handed participants and asked all of them to bring a same sex significant other with them, enabling the social presence condition (for a definition of the significant other see Section 2.3 Data Collection). The findings of the current study include the results of 19 participants (N = 19; females = 11, males = 8), as 11 data sets were excluded due to too many artefacts (i.e., eye blinks, movements) within the EEG recording, invalid measurements of SCR, or invalid answers in the self-reported arousal (i.e., an answer below one or above seven (see Section 2.2. Stimuli)). Apart from incorporating valid measurements within each of the three parameters, all data sets included in the results further had to be valid and without too many artefacts within both of the two conditions. Due to this multi-methodological approach, this number has increased more than initially assumed, as only participants with clean data sets regarding all of the three measures (EEG, SCR, and self-report) were included. All participants were between the age of 18 and 35 years old and most were enrolled in either secondary or tertiary education programs. All participants reported to have normal or corrected to normal vision, no current health issues including no history of psychopathologies, and not to have taken any mind-altering drugs or medications within the past three days. The study was approved by the ethics committee of the Webster University in Saint Louis, MI, USA.

### 2.2. Stimuli

The emotion-related pictures were presented to the participants for 1000 ms on a Dell E2214hb 21.5” widescreen LED LCD monitor with black background. The experiment was designed with the E-Prime 2.0^®^ software package (Psychology Software Tools311 23rd Street Ext., Suite 200, Sharpsburg, PA, USA). All pictures included in the study were retrieved from the OASIS database and were pre-rated in the dimensions of valence and arousal on a 7-point Likert-scale (i.e., 1 = very unpleasant to 7 = very pleasant for valence; 1 = not arousing at all to 7 = very arousing for arousal) [27,28]. To establish two on average equally pre-rated sets of pictures (i.e., alone condition and significant other condition) with 90 pictures per condition (i.e., 30 pictures per emotion category—pleasant, neutral, and unpleasant) the whole 900 pictures found in the OASIS database were firstly categorized into pleasant, neutral, and unpleasant pictures. Pictures with pre-valence ratings from 1 to 3.5 were assigned to the category of unpleasant pictures, images rated from 3.51 to 4.5 were allotted to the category of neutral pictures, and pictures with ratings ranging from 4.51 to 7 were ascribed to the category of pleasant pictures. To ensure valid measurement between the conditions concerning the valence dimension and avoid biases due to high differences in the arousal elicited by the pictures, two highly similar sets of pictures with regards to the arousal dimension were established. Hence, all selected pictures across the three mentioned categories were within the range of 3.74 to 5 on the dimension of arousal. As for each of the two conditions 30 pictures per valence category were needed, overall 60 pictures per valence category were selected. Given that more than 60 pleasant and unpleasant pictures with arousal ratings of 3.74 to 5 were found, the 60 pictures with the highest valence rating of the pleasant pictures and the 60 pictures with the lowest valence rating of the unpleasant pictures (i.e., the ostensibly most pleasant and unpleasant pictures) were chosen.

To achieve the most similar overall valence means within both categories, the 60 pictures selected for each valence category were—on the basis of their respective valence rating—evenly divided into two sets of pictures, each designated to one condition of the experiment. The mean arousal was 4.35 (SD = 0.36) for the unpleasant condition, 4.28 (SD = 0.29) for the neutral condition, and 4.27 (SD = 0.31) for the pleasant condition. *T*-test results between the picture categories revealed no significant difference in the arousal values with a *p*-value of *p* = 0.12 (t(59) = 1.56) for the pleasant and unpleasant pictures, *p* = 0.18 (t(59) = 1.35) for the neutral and the unpleasant pictures, and *p* = 0.79 (t(59) = 0.27) for the neutral and pleasant pictures. This ensured minimal biases due to arousal differences between the picture categories. The *t*-test results of the valence dimension showed clear significant differences between the picture categories, justifying their division into valence categories with all *p*-values < 0.001 (unpleasant-neutral: (t(59) = −24.15), unpleasant-pleasant: (t(59) = −62.1), and neutral-pleasant: (t(59) = −101.07)). The mean valence value of each category shows that the pictures were on average situated around the median of each category, with the mean valence of the unpleasant pictures 2.02 (SD = 0.35) (on the scale rated as 1–3), of the neutral pictures 4.04 (SD = 0.31) (on the scale rated as 3.5–4.5), and of the pleasant pictures 6.08 (SD = 0.16) (on the scale rated as 5–7). This enabled two highly similar pre-rated sets of pictures with regards to their valence and arousal dimensions.

One trial (i.e., all shown events before an emotion picture presentation, picture presentation itself and all events after picture presentation) consisted of a blank screen (1 s), a fixation cross (1 s), a second blank screen (1 s), the respective picture attributed to the trial by the software (1 s), a third blank screen (1 s), and, finally, a valence and an arousal rating screen. Due to complications in the programming, valence ratings were given by the participants but not recorded by the software. Consequently, they could not be evaluated in the present study, resulting in the use of solely the collected and recorded arousal data.

The latter screens were set to have indefinite duration time with its termination dependent on the time of the response of the participants. During the second blank slide baseline activity was measured. The actual measurement of the ERPs in response to the pictures was conducted during the third blank slide, one to two seconds after the onset of the picture and up to one second after the picture was shown. Self-report was requested two seconds after the onset of the stimulus and one second after the picture had vanished. The fixation cross was included to help participants fixate their glance on the middle of the screen to avoid ocular artifacts during measurement. Further, the second during which the fixation cross appeared was the chance for participants to blink outside of the measurements.

### 2.3. Data Collection

The “Inclusion of the Other in the Self” (IOS) Scale was used to approximate the subjective degree of closeness in the relationship of the main participant and their significant other [29]. The second participant was considered as a significant other when the pair of participants rated their closeness to be 5 or higher on the 7-point Likert scale of the IOS Scale. To accurately measure self-reported arousal in response to the pleasant, neutral, and unpleasant pictures the current study used another 7-point Likert scale with one representing very low arousal and seven indicating very high arousal [28].

The participant’s brain activity was measured through EEG. The data was collected using the Geodesic EEGTM System 400 with a silver chloride HydroGel Geodesic Sensor Net of 64 electrodes. While applying an online bandpass filter from DC to 30 Hz, all potential changes were constantly sampled at the rate of 1000 Hz through the EGI Net Amps 400 amplifier with a built-in Intel chip. The data was obtained by means of the Net Station 5.4 software.

SCR was recorded with a Nexus-10-SC/GSR sensor consisting of two electrodes, one attached to the middle finger and the other to the ring finger of the participant’s left hand. The data were acquired with a Nexus 10 wireless recording device (from Mindmedia) and recorded with the Bio-trace+ software.

### 2.4. Procedure

Upon arrival at the CanBeLab (Cognitive & Affective Neuroscience and Behavior Lab) of Webster Vienna Private University, the participants were introduced to the purpose of the study, received information concerning its methodology, and how to reduce potential signal artifacts to ensure good data quality. The experimenter then handed the informed consent form to the participants, explained it orally and asked the participants to sign it upon agreement to participate in the study. The participants were informed that they were allowed to decline their participation or withdraw from the experiment at any point in time without any negative consequences. To determine the closeness of the relationship of the two participants, the experimenter asked the participants to fill out the IOS survey together. The EEG sensor net was applied with all electrodes connected to the ground and referenced to the Cz point. Impedance was kept below 50 kΩ. With the EEG set in place, the experimenter connected the electrodes of the Nexus device and ensured that the participant was seated 65 cm away from the computer screen.

The participants were instructed to indicate their respective rating of every picture by pressing the corresponding numbers from one to seven on the keyboard in front of them. They were asked to answer as intuitively as possible, so as to diminish the interference of cognitive influence and approximate affective responses. The participants were instructed to remove all electronics on them to reduce artifacts, to stay as still as possible—implying an abstinence from talking, blinking, or swallowing—, especially shortly before, during, and after the stimulus presentation. After the experimenter started the visual presentation of the pictures via the E-prime 2.0^®^ software, the participants had time to thoroughly read the instruction screen and start with the experiment as soon as they were ready by pressing the spacebar on the keyboard in front of them. In the meantime, the experimenter made sure Biotrace+ was ready for recording and started the recording session as simultaneously as possible with the participant pressing the spacebar.

After the first condition had been concluded, the second participant (significant other) was asked to enter the room and stand next to the main participant. The significant others were instructed to position themselves so they would see the face of the main participant as well as the screen with roughly one arm length between the two participants. The participants were told they should maintain the feeling of watching the pictures together as they would normally do at home, facilitating immediacy and intimacy as defined by [30].

To avoid artifacts in the EEG signal through eye or head movements by the participants, we ensured for the pictures to be viewed fully with a straight stare, wherefore they were reduced in size to be merely presented in 75% of width. The part of the screen which did not show the picture, remained black to avoid further light stimulation or attention distraction. To prevent carry-over effects between the respective valence categories, a block design for picture presentation was chosen, showing the 30 pictures of each valence category successively in one go without mixing valence categories.

To avoid order effects, the blocks were sequenced, and the participants watched the pictures either first alone or first with the significant other on alternating turns (i.e., participant number one watched the sequence of pictures in the order pleasant–neutral–unpleasant pictures (PNU), participant number two in the succession neutral–unpleasant–pleasant (NUP), and participant number three in the sequence unpleasant–pleasant–neutral (UPN), and so forth). Within each valence block, the presentation of the pictures was randomly displayed by the software. In addition to this change in sequence in the picture categories, the order of the two highly similar picture sets was counter-balanced to control for any undesired order effects. Participant number one to three would all start with picture set number one while participants number four to six would all start with picture set number two (and so on). Hence, participant one would start with picture set one and watch the sequence of pictures PNU, participant number two would also start with picture set one and watch the sequence of pictures NUP, and participant three would start with picture set one and watch the sequence of pictures UPN. Participants four to six would all start with picture set two and while participant number four would watch the sequence PNU, participants number five and six would watch the order NUP and UPN, respectively. As after six participants all sequences are counterbalanced, participant number seven would be subject to the same picture sequence and starting picture set as participant number one.

To control for order effects related to the presence of the significant other, participant one to six would all first watch the sequence of pictures alone—no matter what picture set and valence category would start—and participants seven to twelve would start watching the sequence of pictures with the significant other being present. This method was implemented for all participants.

### 2.5. Data Analysis

To process and clean the EEG data, EEGDISPLAY 6.4.9, a custom-made software by Ross Fulham, was used. All data sets were subject to an offline bandpass filter from 0.1 to 30 Hz. Subsequently, baseline-corrected epochs were created and all data from 100 ms before the onset of each stimulus to 1000 ms post stimulus onset were extracted. The resulting epochs were then visually inspected, and any visible artifacts or amplitudes marked by the software as over 75 mV were removed by an automated threshold detection software. The ensemble average for all electrodes was re-referenced to the common average and in the end grand averages of all 19 (valid) data sets were established for both the alone and the significant other condition. For statistical analysis, paired-samples *t*-tests were calculated for a selection of two frontal electrode locations (electrode 6, which is around AFz and electrode 8, which is around FPz) to compare respective amplitude means of single potential values 708 ms post-stimulus onset between both conditions. The electrode locations were selected on the basis of their known sensitivity to ACC activation changes [31]. In order to conduct source localization all electrode data were used to run low resolution electromagnetic tomography (LORETA) [32].

The E-prime 2.0^®^ software also recorded self-reported arousal answers. The data were categorized according to their valence category and condition. Mean reported arousal and SD were computed per condition for every category. Paired sample *t*-tests comparing the results of the significant other and the alone condition were conducted between all picture categories.

SCR was measured across the entire run for each condition (alone and a significant other) without differences in the valence categories of the pictures. Biotrace+ measured arousal differences with a sample rate of 32 samples per second (SPS). All arousal data were exported from Biotrace+ and averaged separately for both conditions per participant. The SCR value for the significant and the alone condition was averaged, respectively, and SD was calculated. Statistical significance between the two means was determined through a two-sided paired sample *t*-test.

Assuming higher arousal in the significant other condition than in the alone condition, the averaged result of the alone condition was subtracted from the averaged result of the significant other condition. This was performed separately for every participant. In the end, all results, representing each participant’s difference between the arousal value in the significant other and the alone condition were averaged for the final result.

## 3. Results

### 3.1. EEG Results

**Figure 1:**
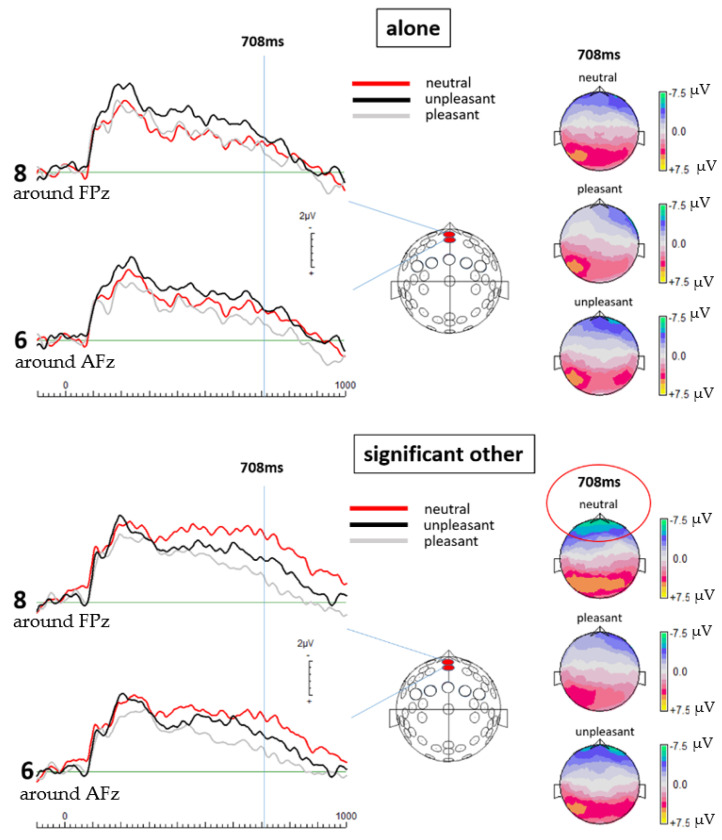
On the left: ERPs from both selected electrode locations for all three emotion categories and both experimental conditions are shown. On the right: Topographical maps for each valence category and both experimental conditions are shown. The ERPs and their topographic display show clearly that neutral pictures in the significant other condition elicit the highest levels of brain activation at mid-frontal electrode locations, also emphasized by the top topographical map in the significant other condition (circled in red color). The topographical maps further show cortical activity elicited in the significant other condition to be constantly more negative than in the alone condition, regardless of the valence category. This further emphasizes to what extend social presence alters human brain activity and consequently, most likely also resulting decision-making mechanisms.

Contrasting the respective ERPs, data processing revealed significantly more negative going potentials at the selected central frontal electrode locations when participants were exposed to the neutral pictures while with a significant other versus when alone. The most significant difference in activation was found at 708 ms post stimulus onset, as revealed by multiple paired *t*-test at different points in time (see Figure 1 and Table 1). Although not reaching statistical significance, slightly more negative going potentials were also detected for the unpleasant and pleasant pictures when the participants were with a significant other than compared to alone (see Figure 1 topographic maps). Paired sample *t*-tests, comparing the ERP results between the valence categories (unpleasant, pleasant, and neutral pictures) of each electrode revealed significant differences.

At 708 ms, results of the alone condition showed no significant differences in the cortical activation between the separate valence categories. No significant difference was found for the unpleasant and pleasant pictures with a *p*-value of 0.236 (t(18) = 1.225) for electrode 6 (around AFz) and a *p*-value of 0.376 (t(18) = 0.908) for electrode 8 (around FPz). Further, no significant difference was detected between the neutral and pleasant pictures for electrode 6 (around AFz), *p* = 0.345 (t(18) = −0.969); for electrode 8 (around FPz), *p* = 0.673 (t(18) = −0.429), as well as between the neutral and unpleasant pictures with a *p*-value of 0.625 (t(18) = 0.497) for electrode 6 (around AFz) and 0.647 (t(18) = 0.466) for electrode 8 (around FPz). Although not statistically significant, differences in cortical activation between the valence categories are indicated by the graphical display of the ERPs as well as the topographic maps (see Figure 1) reflecting unpleasant pictures to elicit the strongest ERPs overall in the alone condition.

In the significant other condition, *t*-test results from electrode 6 (around AFz) and 8 (around FPz) at 708 ms showed a significant difference between the neutral and pleasant condition with *p* = 0.02 (t(18) = −0.2551) for electrode 6 (around AFz) and *p* = 0.007 (t(18) = −3.063) for electrode 8 (around FPz). Further, a significant difference was detected between the unpleasant and the pleasant pictures at electrode 8 (around FPz) with *p* = 0.02 (t(18) = 2.544). A strong trend was found at electrode 6 (around AFz) between the unpleasant and pleasant condition with *p* = 0.095 (t(18) = 1.760) as well as at electrode 8 (around FPz) between the neutral and unpleasant condition with *p* = 0.098 (t(18) = −1.747). A non-significant difference was found between the neutral and unpleasant condition at electrode 6 (around AFz) with *p* = 0.115 (t(18) = −1.654). Hence, when with a significant other, neutral pictures elicited the most negative going potentials at the selected mid-frontal electrode locations. The *t*-test results at both electrode sites reflect a strong difference in the cortical activation between the neutral and the pleasant condition. Interestingly, although no statistical difference is detected at both electrode sites between the neutral and the unpleasant pictures, the results of electrode 6 (around AFz) reflect a strong trend of a difference between these conditions, indicating unpleasant pictures to still trigger the second strongest activity in the brain.

**Figure 2:**
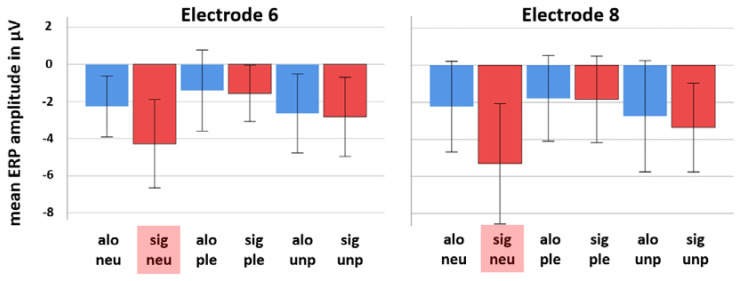
Bar diagrams (including standard deviations) showing mean ERP amplitudes at 708 ms post-stimulus of all emotion categories and both social conditions (alone (alo) and significant other (sig)) for electrode 6 and 8. Note that the condition “significant other neutral emotion category” (sig neu, marked in red color) constantly elicited the most negative going brain potential at both electrode locations with a larger effect at electrode 8 (around FPz). Significant other pleasant = sig ple, significant unpleasant = sig unp, alone neutral = alo neu, alone pleasant = alo ple, alone unpleasant = alo unp.

Figure 2 shows bar diagrams of mean amplitudes at 708 ms post-stimulus of all emotion conditions and both social conditions (alone and significant other) for electrode locations 6 (around AFz) and 8 (around FPz).

**Figure 3:**
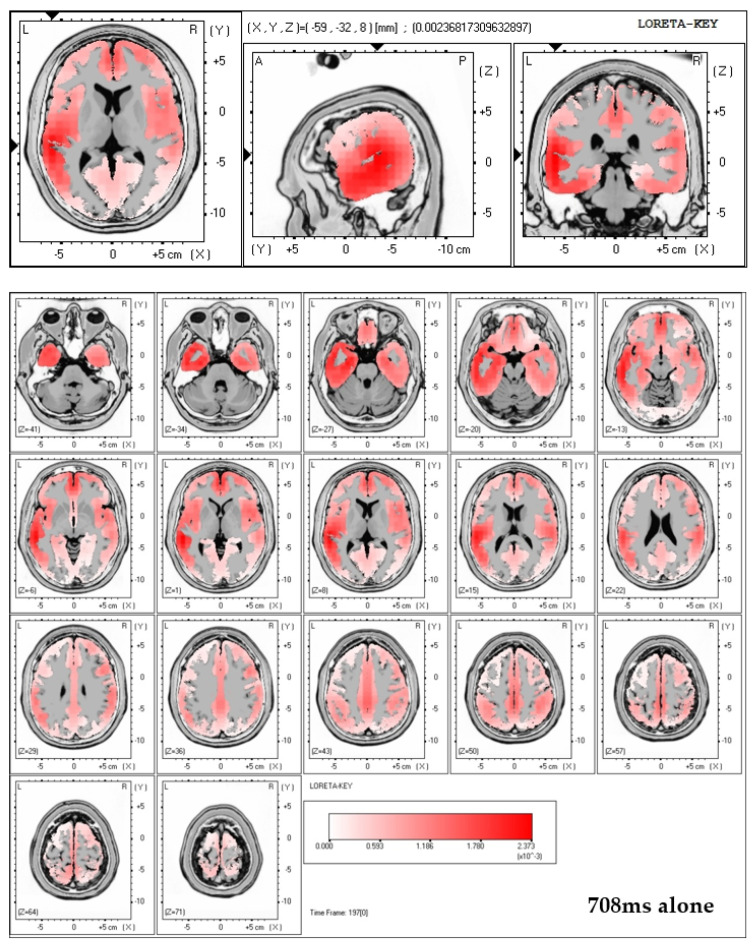

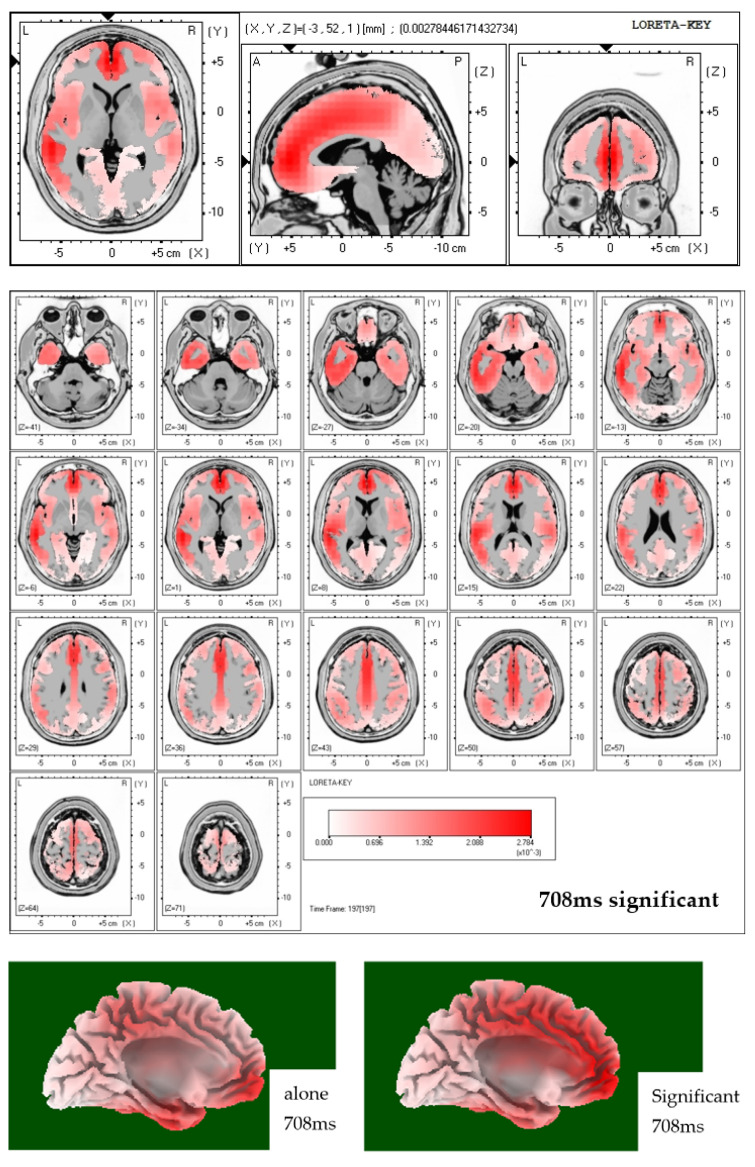
Top two figures: LORETA solutions including all 64 electrodes for both conditions (alone and significant) at 708 ms post-stimulus onset in case of neutral pictures. Note that in the alone condition LORETA calculated maximum activity in the left-temporal region. In the significant other condition, exposure to neutral pictures resulted in maximum activity in the central, frontal region, which can be interpreted as originating within the ACC. Bottom figure: three-dimensional brain displays showing that LORETA solutions calculate maximum brain activation in the significant other condition while viewing neutral pictures to reside within the ACC region.

Finally, LORETA solutions show that the exposure to neutral picture while being accompanied by a significant other indeed elicits stronger brain activation in the region of the ACC (see Figure 3).

### 3.2. Self-Reported Level of Arousal

**Figure 4:**
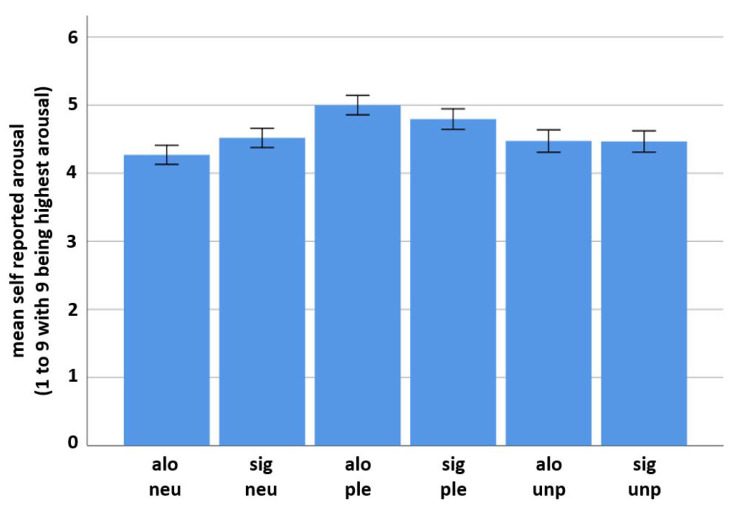
Self-reported arousal values (including standard deviations). Significant differences between both social conditions were found for the neutral and the pleasant, but not for the unpleasant emotion category (see above for *t*-test results). In the neutral emotion condition, the presence of a significant other person resulted in higher self-reported arousal, whereas in the pleasant emotion condition being alone resulted in higher self-reported arousal. For variable names see Figure 2.

Self-report data also reflect a valence-specific difference in the subjective perception of arousal. Mean reported arousal for the alone-neutral condition was 4.27 (SD = 1.71), for the alone-pleasant condition it was 5 (SD = 1.74) and for the alone-unpleasant condition it was 4.47 (SD = 1.998). Mean reported arousal for the significant other-neutral condition was 4.52 (SD = 1.72), for the significant other-pleasant condition it was 4.8 (SD = 1.83) and for the significant other-unpleasant condition it was 4.47 (SD = 1.9) (see Figure 4). *T*-test results revealed a significant difference between both social conditions for the neutral emotion category (t(18) = −2.724), *p* = 0.007) (significant > alone) and also for the pleasant emotion category (t(18) = 2.668), *p* = 0.008) (alone > significant). No such difference was found for the unpleasant emotion category (t(18) = 0.078), *p* = 0.938).

### 3.3. Level of Arousal as Measured with SCR

**Figure 5:**
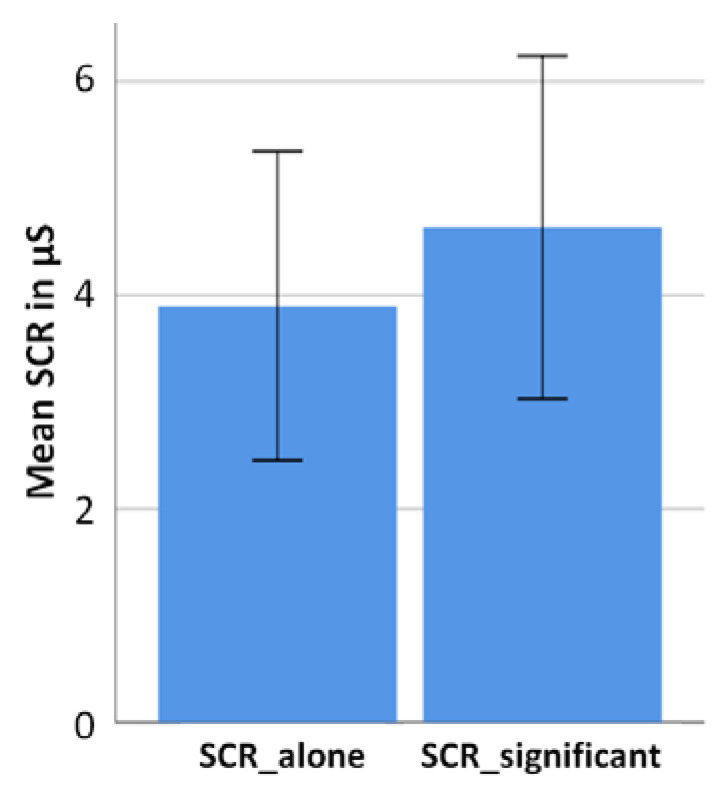
Bar diagram (including standard deviations) showing mean SCR values across all participants (*N* = 19) for both experimental conditions (alone and significant other conditions). The arousal level is significantly higher in the significant other condition compared to the alone condition.

Measurements of the sympathetic nervous system were not tailored to valence-specific categories but measured across the entire social conditions (alone versus significant other). The averaged SCR value for the alone condition was 3.9µS (SD = 3) and for the significant other condition it was 4.6 µs (SD = 3.3). The difference between these two means is significant (t(18) = −2.612, *p* = *0*.018). Figure 5 reflects the individual arousal values of the participants in the significant other condition and the alone condition. Thus, SCR measurements reflect similar results when compared to EEG and self-report data, implying the presence of a significant other to have a potentiating impact on all of the three the measured parameters.

## 4. Discussion

Despite an array of findings, demonstrating that social presence has an impact on human feelings linked to emotion-related and cognitive processing [33,34,35,36,37], its influence on brain structure and function [38], as well as on neurocognitive mechanisms of decision-making [39,40,41,42,43] has only moved more into the focus of research within the recent decade. The results of the current study show that social presence, in form of the presence of a significant other, has an impact on neural activity related to affective processing, reflected in human brain activation and SCR, as well as in subjectively perceived arousal, mirrored in self-report. Beyond that, the present results indicate a close relationship between cerebral cortical activity and sympathetic arousal, as implied by Lim et al. [25]. The current study demonstrates such an interrelation with respect to the presence versus absence of a significant other. This is indicated by an elevated level of neural activity and sympathetic arousal in the significant other condition compared to the alone condition when viewing emotion-related pictures. Strikingly, this was only found for neutral pictures, which might indicate that the presence of a significant other challenges ambiguous emotion ratings most dominantly. Although not of statistical significance, EEG measurements further mirrored neural activity to be the strongest in response to unpleasant pictures when alone.

### 4.1. Brain Structure and Function in Relation to Social Stimuli

Assuming that our EEG findings indeed reflect ACC activity changes, we intend to further interpret them in that direction. In humans, evidence from brain imagining studies, including electroencephalography (EEG), suggests the anterior cingulate cortex (ACC) to represent an integrative weighting instance responsible for the monitoring of adaptive social cognition mechanisms [5,10,23,44,45,46,47,48]. The ACC adjusts the identity and intensity of control signals to reach large overall incentives, as its vital function includes control-signal specification of generally aversive signals [3,11,23]. This multifunctional key encoding role of the ACC has been demonstrated in studies conducted within a variety of decision-making contexts, such as conflict and error detection [12,44,45], outcome monitoring [13], cognitive effort [14], defense and attack strategies [15], financial losses [16], experienced and observed pain [17], socially-driven interactions [8], empathy-related responses [12,17,49], as well as updating internal beliefs and environmental estimates in the decision between alternative strategies of action and the default option [5].

Rudebeck et al. [43] were further able to demonstrate that despite the fact that integrative signals in a variety of brain areas are responsible for the processing of social information, as well as the consecutive integration of social behavior, the ACC seems to have a decisive role in mediating value associated with social stimuli. This crucial role of the ACC in normal social engagement with conspecifics is underpinned by the fact that the presentation of non-social control objects did not cause similar ACC activity compared to social stimuli [43].

### 4.2. Neurocognitive Mechanisms of Social Cognition and Decision-Making in the ACC

Evidence from neuroimaging and other electrophysiological experiments indicate a further role of the ACC in integrating affective as well as high-level cognitive processes linked to economic, social, and survival-related decision-making. Within this context, the ACC is suggested to be involved in the human (social) pain network [40,41,50,51] along with the neural computation of choice value related to as many topics as action selection [52,53], effortful decision-making [14,18], cost-benefit estimations in weighting alternative strategies [5], motivational factors in oneself and the social counterpart [1,54], as well as the calculation of the expected value of control [3,55]. The latter refers to the computation of an estimate of the needed adjustment and signal-specification of control, required to adequately determine one’s own as well as others’ motivation to engage in a certain task, including factors of the current social environment [1,3,6,41,50,54,55].

Investigating the effects of ACC lesions on the neural computation of action selection, Thaler et al. [53] found that monkeys, whose ACC had been removed, had severe difficulties in instigating actions. Studies conducted with patients suffering from ACC damage, further report an almost absolute absence of goal-directed behavior [52]. In rats, inactivation of the ACC was linked to diminished willingness to engage in decision-making that requires either cognitive or physical effort [14]. Further, increased metabolic activity in the ACC complex of rats was noticed during tasks, which involved changes of effort costs and reward magnitude compared to tasks where solely reward magnitude was alternated but effort costs remained steady [14]. A link between high mental effort decision-making and ACC activation was further reported in humans by Mulert et al. [18].

Centered in evidence from neurophysiological and neuroimaging studies, another integrative account of ACC function explicitly describes a key role of the ACC in social cognition, while resembling Kolling et al.’s. and Botvinick and Braver’s theories [5,54]. Unifying the multidimensionality of ACC function, this theory specifically proposes the ACC’s main functions of detecting and monitoring costs, benefits, and errors in social context to represent a larger pattern of affective and high-level cognitive processing during social interaction [1]. Corresponding to the neural correlates of decision-making mentioned by Kolling et al. [5], Apps et al. [1] suggest the ACC to deliver a major contribution to the accurate estimation of the level of motivation of a social counterpart and continuously updating them, when stimuli imply that previous estimates have been inaccurate. Adequately recognizing the motivational level of another individual is of vital significance, since it can provide important information about the cognitive and affective states of others, as well as the subjective value of an option, and thus, potential behavior of others can be predicted [1,15]. This calculation is referred to as the vicarious value (vV) of motivation, or “the value of behavior for another”, which is important for an individual to predict the potential future actions of others and prepare to respond in the most adaptive way [1]. In line of all abovementioned ACC activities and functions, we interpret our ERP findings as being reflective of ACC involvement in emotion-related information processing with a focus on neutral emotion content during the presence of a significant other person.

### 4.3. Interpretation of the Present Results

Even though the inverse problem—a problem not unfamiliar to the methodology of EEG—does not allow for a direct source localization, inferences about the neural source of the measured cortical activity may be possible through source localization techniques and the grounding of assumption on studies attributing activation to similar electrode locations. A substantial amount of such neuroimaging studies have assumed the ACC to be associated with electrode locations 6 and 8 and their respective positioning [10,12,13,31,42,45,50,51,56]. LORETA solutions applied to the ERP-data of this study indeed showed increased activation (activation maxima) in the ACC region in case of viewing neutral pictures while being accompanied by a significant other.

Within this context, a solid body of literature demonstrates the involvement of the ACC in various aspects that are investigated in this study. Examining medial prefrontal cortex activation (mPFC) in relation to appraisal of ambiguous affective stimuli, Simmons et al. [10] found significantly more ACC activation in ambiguous trials compared to unambiguous trials, as well as in trials involving ambiguous affective decisions than in trials requiring ambiguous gender decisions. This corresponds to the results of the current study in the significant other condition, where the neutral, hence ambiguous affective pictures elicited the strongest ERPs at electrode locations 6 and 8. While Botvinick [23] suggest the ACC to monitor and detect any aversive signals within the incoming stream of information, another study reported ACC activation to not only occur within several qualities of uncertainty and ambiguity processing but further observed activation patterns of—inter alia—the ACC to mirror individual uncertainty attitudes [24]. Further studies report increased ACC activation in response to unpleasant affective stimuli [57], as well as negative affect and pain processing [9,22]. In our study, no significant effects were found in case of negative emotion pictures.

Heightened ACC activation, as measured with fMRI, has also been linked by previous studies to autonomic arousal, measured through electrodermal activity, hence implying a role of the ACC in the intentional modulation of physiological states of arousal [58]. This function of the ACC is consistent with its role in the regulation of output signals related to the execution of behavioral responses [59]. The results of the present study provide further evidence for this relationship, as our measurements were able to demonstrate significant activation in the area of the ACC, while simultaneously recording sympathetic arousal and self-report data in line with the measured cortical activity. Self-report data are understood as an indicator of conscious appraisal as well as conscious thought. Thus, the recorded ERPs in the present study are interpreted to reflect cortical activity responsible for the adequate adjustment of the signals, while sympathetic arousal and self-reported arousal are assumed to mirror neural projections that control the resulting behavioral response. The combination of present results with the findings of those and more studies, further, allow for an interpretation about the overlapping ACC activity similarly to one made by Dalgleish et al. [40], namely that the ACC plays a decisive role in gauging relevant (social) stimuli and consecutively, mediates this information to other substrates responsible for the execution of control in relation to an individual’s internal and environmental circumstances. This monitoring with regards to the individual’s social inclusion status explains why the subjects of the current study displayed the most heightened activity when confronted with neutral (thereby ambiguous) stimuli while in social presence.

## 5. Conclusions

Concluding, the most striking finding of the present study is that social presence strongly affects neural activity of affective processing in case of the neutral emotion condition to an extend that all three parameters of cortical activity, sympathetic arousal, and self-report, as measured in this study with EEG, SCR, and subjectively reported arousal, are affected in the same direction. No such effects were found for the pleasant and also not the unpleasant emotion condition. Our source localization results indicate the ACC to show increased activity levels depending on social presence in the neutral emotion condition. As abnormal function of the ACC was related to a number of psychiatric disorders [12] and functional changes in the brain structure of a neural network, involving the ACC, “may be partially a consequence, and not just the cause, of alterations in social interactions” [38], this is becoming ever-more important in an age coined by increasingly individualistic societies.

For an exhaustive comprehension of the extend and identity by which social presence influences affective and cognitive processing, more research is needed. Concretely, it would be interesting to investigate if all social stimuli and social contexts, elicit similar patterns of cortical and sympathetic activity, as well as subjective perceptions. Due to the complexity of social life and its implications for survival and well-being, it is likely that such neural patterns are dependent upon a variety of factors, such as the degree of sociality of a social stimulus, the availability of information about the other’s social background [42], and whether the interaction takes place with an individual from an in-group or out-group [60]. Additionally, the context of a social situation (i.e., whether it is positive or negative, interfering expectations on both sides, the relationship to the person that provides the social context, individual past experiences, as well as non-social environmental facts) are expected to have an impact on neural activity and its associated mental and bodily functions.

## Figures and Tables

**Table 1:** *p*-values of ERPs at electrode 6 and 8 708 ms post stimulus onset between the respective valence categories in the alone and the significant other condition. Significant values are marked in red. Trends are marked in orange.

Alone	At 708 ms	
	Valence Categories	*p*-Value
**Electrode 6** **(around AFz)**	Neutral-Pleasant	*p* = 0.345 (t(18) = −0.969)
	Neutral-Unpleasant	*p* = 0.625 (t(18) = 0.497)
	Unpleasant-Pleasant	*p* = 0.236 (t(18) = 1.225)
**Electrode 8** **(around FPz)**	**Neutral-Pleasant**	*p* = 0.673 (t(18) = −0.429)
	Neutral-Unpleasant	*p* = 0.647 (t(18) = 0.466)
	Unpleasant-Pleasant	*p* = 0.376 (t(18) = 0.908)
**Significant other**	**At 708 ms**	
	**Valence Categories**	***p*** **-Value**
**Electrode 6** **(around AFz)**	Neutral-Pleasant	*p *= 0.020 (t(18) = −2.551)
	Neutral-Unpleasant	*p* = 0.115 (t(18) = −1.654)
	Unpleasant-Pleasant	*p* = 0.095 (t(18) = 1.760)
**Electrode 8** **(around FPz)**	Neutral-Pleasant	*p *= 0.007 (t(18) = −3.063)
	Neutral-Unpleasant	*p* = 0.098 (t(18) = −1.747)
	Unpleasant-Pleasant	*p *= 0.020 (t(18) = 2.544)
